# Does Losing on a Previous Betting Day Predict How Long it Takes to Return to the Next Session of Online Horse Race Betting?

**DOI:** 10.1007/s10899-020-09974-x

**Published:** 2020-09-18

**Authors:** Tuomo Kainulainen

**Affiliations:** Department of Business, University of Eastern Finland, Joensuu Campus, P.O. Box 111, 80101 Joensuu, Finland

**Keywords:** Online gambling, Consumer behaviour, Loss-chasing, Panel data, Empirical, Statistical, Horse racing

## Abstract

This study examined how recent losses predict the frequency of play in online betting. Previous studies have suggested that players tend to decrease betting volume and consequently take on less risk after a losing session. We used a daily panel of actual gambling data and survival regression to investigate how incurring losses predicts the duration to the next betting day. Our main findings were that, after a losing betting day, a bettor typically abstained from betting for a 27% longer time than after a day he or she broke even. Further, we found that either untypically high wins or loses significantly predicted the amount of time to the next gambling event. This study adds to the gambling literature by presenting evidence on a reduction in betting activity following a losing session.

## Introduction

While individual-level gambling account data is a fairly recent phenomenon in gambling research, studies employing such data have contributed to a better understanding of gambling behaviour in various contexts. Early work provided overviews of online players’ behaviour over time in gambling forms such as sports betting, casino and poker (e.g., LaBrie et al. 2007, 2008; LaPlante et al. 2009). These contributions used cross-sectional gambling data and behavioural metrics, which allowed comparisons between, for instance, the representative bettor’s daily betting volume or betting frequency in diverse gambling activities. This lead to a better understanding of behavioural differences between the forms of online gambling.

More recently some empirical gambling studies have used player account data to study how prior losses predict subsequent betting behaviour. These studies may analyse either within- or between-session behaviour. Within-session studies investigate players’ immediate reactions to losses in terms of whether and how much they bet during their current session. Between-session work focuses on whether and how soon players return to gambling after losses. Interest in between-session behaviour is linked to problem gambling screens, such as DSM-V, asking whether the individual returned to gambling another day to try to recover losses.

Narayanan and Manchanda (2012) studied betting behaviour within ‘a trip’ to the casino. They analysed a random sample of 2000 loyalty card customers whose gambling behaviour was observed over 2 years at a particular casino. The majority of betting in their data comes from slots and the median length of contiguous play within a ‘trip’ lasts around 2 h. Narayanan and Manchanda (2012) found that over 95% of casino gamblers were less likely to continue gambling if they had won on their previous bet. They also noticed that average casino gamblers tended to keep on playing when losses or wins were small. However, gamblers quit the session when their cumulative losses or wins exceeded a certain high amount. Narayanan and Manchanda (2012) interpret their findings as constituting evidence of belief in the gambler’s fallacy [i.e. gamblers are less likely to bet money if they have just won money and vice versa] among a significant proportion of consumers.

Suhonen and Saastamoinen (2018) studied how prior betting outcomes predict current consumption within a session using Finnish online horse race betting data. They focused on a horse race meeting in August 2012, modelling *race*-*to*-*race* betting behavior of 5217 online bettors. The present paper analysed *day*-*to*-*day* betting behaviour for 30 days and this period includes the fixture which Suhonen and Saastamoinen (2018) focused on. They found that bettors tended to decrease their betting volume in the next race by 4% if they had incurred losses in prior races. If a bettor was ahead, however, it predicted a 6% increase in betting volume in the next race.

Ma et al. (2014) analysed between-session behaviour with online gambling data from Bwin, a major international gambling operator, using accounts of 22,304 new customers that were followed for 8 months. They used week-level aggregated panel data, which included all gambling types available (i.e., poker, casino games, video games and sports betting). Their objective was to study how players’ cumulative outcomes and recent outcomes in gambling predict players’ behaviour in the next week. They found that customers who lose in the previous week tend to cut their bet sizes by around 26% in the following week.

Forrest and McHale (2016) were the first who used survival regression to study between-session loss-chasing behaviour by loyalty card holders playing slot machines at Rank Group casinos over a 4 year period. According to their definition, loss-chasing behaviour was present when a player returned to play after an atypically high losing session sooner than otherwise. Since their empirical model required that an individual engages in multiple consecutive betting sessions, they restricted the analysis to regular gamblers who made at least 50 visits to a casino in a year. They found that for most players, being a loser on the last visit predicts a longer time to the next visit. However, around 2% of players had a tendency to return to play sooner than usual after an atypical loss, which they regarded as an indication of loss-chasing behaviour.

To summarise, the main finding of the studies that analysed the association between prior losses and current betting, gamblers tended to reduce their risk in betting after losing the preceding bet or on a losing visit. This indicates that it is unlikely that the representative bettor is a loss-chaser. However, an exception was Narayanan and Manchanda (2012) who found that land-based casino gamblers reacted in the opposite manner to a losing bet in a gambling session. Ma et al. (2014) discussed possible reasons for this contradictory finding. They commented that ‘In particular, Narayanan and Manchanda (2012) conducted a within-trip analysis of casino visits that is based on the assumption that the outcomes of previous trips do not affect subsequent gambling. Such an assumption might be reasonable for casino gambling, but seems unrealistic for online gambling’. However, Suhonen and Saastamoinen (2018) detected that online horse race bettors actually tend to reduce their bets after a losing bet within a gambling session. Thus, the reason(s) for the contradictory result obtained by Narayanan and Manchanda (2012) remains unclear.

This paper adds to a small but growing research area which uses individual account data to study how current gambling consumption is affected by past outcomes of betting. We expand the literature (Narayanan and Manchanda 2012; Ma et al. 2014; Forrest and McHale 2016; Suhonen and Saastamoinen 2018) by analysing how being a loser on the last betting day predicts time to next participation in online horse race betting. Since the previous literature is sparse and contains some contradictory results, more research is needed to assess players’ behaviour after losing betting sessions. The novel contribution of this study is to model a player’s time to the next betting session as a function of recent betting outcomes in online betting.

The early empirical research on individual gambling behaviour over time typically used metrics such as betting frequency or duration of gambling (e.g. LaBrie et al. 2007, 2008; LaPlante et al. 2009), employing cross-sectional data to provide an overview of gambling behaviour over a time period. Our approach is to follow bettors’ choices between daily sessions by using the behavioural metric *time to the next betting day*. This allows us to provide novel insight into how a last losing session predicts time to the next session in online gambling. We also study whether untypically high wins or loses predict the amount of time in days to the next gambling event.

Another extension to the prior empirical gambling literature (i.e. LaBrie et al. 2007, 2008; LaPlante et al. 2009) is that we employ statistical modelling. We use survival regression, which is an infrequently used method in this field. While we focus on how the betting outcome of the last betting day predicts the time to the next betting activity, there are multiple confounding factors which should be taken into account in modelling reengagement in betting. For instance, individuals can have diverse gambling habits (e.g. Jolley et al. 2006) or betting participation on some days of the week may be more frequent than on others. Additionally, since our data contain background variables for each player, such as age and gender, we study an association between these variables and time to the next engagement in betting. A survival model can handle several factors simultaneously and provide more precise evaluation of bettors’ re-entry times. To the best of our knowledge, Forrest and McHale (2016) is the only study that examines tracked gambling data using survival regression. Their analysis relates to the behaviour of customers using loyalty cards to play slots at bricks-and-mortar casinos in Great Britain. This study extends their analysis to a different gambling activity.

In general, research questions concerning how long it takes until an event of interest occurs can be examined using survival modelling. The key concept in the analysis is *survival time*, which Mills (2011) defined as the duration or time until event occurs. Survival analysis has been applied in multiple scientific disciplines, for instance, in many areas of social research (Mas-Verdu et al. 2015; Plank et al. 2008; Kiefer 1988) and medical research (Ishak et al. 2013; Lee and Go 1997). The main benefit of survival models is that they perform better than ordinary regression (OLS) models with deviations from normality in the survival data (see Cleves et al. 2010). This study employs parametric survival models, where the distributional assumptions are made based on the best data fit.

We use online horse race betting data provided by the Finnish betting monopoly company. In contrast to previous studies carried out with this data set (i.e. Suhonen and Kainulainen 2016; Suhonen and Saastamoinen 2018; Suhonen et al. 2018; Saastamoinen and Suhonen 2018; Kainulainen 2019), we constructed a daily panel data set for this analysis, and the unit of analysis is the individual betting account holder betting on a particular date. Further, this study analyses the time aspect of gambling behaviour, whereas other studies exploited this data source to model monetary gambling behaviour.

The rest of the paper is organised as follows. “Methods” section describes the individual-level data set, behavioural attributes and survival regression methods used in the analysis. “Result” section presents descriptive statistics and survival regression estimation results. Finally, “Discussion” section discusses the main findings of this paper.

## Methods

### Sample

This study used data on online horse race bettors in Finland. We analysed the raw data from the Finnish horse race betting monopoly operator Fintoto Ltd (now part of the state lottery organisation, Veikkaus Ltd). The data consist of bets made on Fintoto’s online betting platform between 1 August and 30 August 2012. During this period, online horse race players could bet on races in Finland, Sweden, France and the United States. In total, the number of horse race meetings was 93, spread over 50 different tracks. The betting data included full information on all single-race betting types (i.e., Win, Place, Quinella and Trifecta). However, information on multiple-race betting types (i.e. Pick games and Daily Double) was not available. Kainulainen (2019) described the betting data and the Finnish horse race betting environment in more detail. The present study employs a large data set of 9151 bettors who made nearly 4.2 million bets during the observation period. A typical bettor is a middle-aged male who lives in the city.

We followed the procedure presented in Forrest and McHale (2016) by placing the wins and losses of the bettor’s last betting day in a context. Thus, we had to be able to assess the typical player-specific outcome of a betting day in order to define whether the losses of the last betting day had been particularly high for the player. In order to calculate this typical betting behaviour, we split our 30-day data into two parts. We calculated the typical betting behaviour using the first 15-day period and used the second period to estimate the survival regression model. We also defined the bettors’ *past frequency* of betting, i.e., the number of betting days during this period divided by 15, from the first period. In addition, we calculated the typical outcome of a betting day for each individual, which is the aggregated sum of daily outcomes during the first period divided by the number of betting days during that period. We used this metric to calculate the normalised wins and losses for the individual.

Our focus variables in the analysis were a dummy variable for being a *loser on last betting day*, *losses on last betting day* and *winnings on last betting day*. The dummy variable *loser on last betting day* takes the value one if the bettor’s last betting day was unprofitable (i.e., the amount staked was higher than the winnings received) and the variable equals zero if the bettor’s last betting day ended up break-even or profitable. Next, we introduced the concept of normalised wins and losses, which we used to measure untypically high losses or wins for the bettor on his or her last betting day. In general, normalised wins and losses are positive (negative) if the outcome of the last betting day obtained in the second period has the same (opposite) sign as the bettor’s typical outcome of a betting day. When the bettor has lost 50 euro in the last betting day, but his or her typical outcome of betting is − 20 euro, *losses on the last betting day* equals 2.5. Correspondingly, *winnings on last betting day* equals − 0.50, if the bettor’s last betting day net win has been 50% of his or her typical losses. Normalised wins and losses equals 1 if the losses of the bettor’s last betting day were exactly the same amount as his or her typical outcome of a betting day.

The control variables in the analysis included each bettor’s typical play (i.e., *past frequency*). Since there are differences between different days of the week in how popular races are, which are described below, we also controlled for the day of the week. Additionally, the model controls for the bettor’s *age*, gender (i.e. *female*), *urban residence* and *experience* as a bettor. *Urban residence* equals 1 when a person lives in a city and 0 otherwise. We defined the *experience* variable using the operator’s player identification number to divide bettors into high (= 1) and low (= 0) experience bettors. The identification numbers were given in a chronological order when the betting accounts were registered and thus high experience refers to a longer history of being a customer of the betting company and vice versa. Further, the analysis also included the following interactions (1) between *age* and *loser on the last betting day* (*i.e. age* × *loser on last betting day*), (2) between *experience* and *loser on the last betting day* (i.e. *experience* × *loser on last betting day*). We included these interactions to find out, whether, for instance, less experienced bettors reacted differently to being a loser on the last betting day than more experienced bettors.

Since our focus variables were lagged by one time period (e.g. *loser on the last betting day*), we dropped some of the least active bettors from the data to avoid missing values on key variables. We excluded bettors who did not wager in at least one betting day in the first period and at least in two betting days in the second period.^1^ For the remaining 9151 bettors, we constructed a daily panel data set and calculated the variables at the daily level for each bettor. These 9151 bettors generated 55,313 betting days, which we analysed with the survival regression method.

The panel data set had two main limitations. First, it lacked socio-economic variables (e.g. income, education), which would have allowed more detailed modelling. Second, we had only a 30-day period data available for this study. Some bettors may not have participated at all during the timeframe. It is also possible that the betting behaviour recorded may not have represented the typical betting behaviour for some bettors who did participate during the observation period. Moreover, due to the time span, we were unable to fully replicate the analysis of Forrest and McHale (2016) who were able to apply the model at the level of each individual, allowing identification of loss-chasers.

### Survival Regression Method

Survival and event history analysis is an umbrella term for a collection of statistical methods which focus on questions related to duration until occurrence of an event (Mills 2011). The choice of appropriate model is based on the research question of interest and the data available.

In survival or time-to-event analysis, there are two quantities of interest to define. Here we followed the definitions used in the survival analysis literature. The first quantity is the survival function, denoted by *S*(*t*), which provides the probability of survival at a given time. The second quantity is the hazard function, denoted by $h(t) = S^{{\prime }} (t) /S(t)$. Thus, the faster the survival function decreases, the higher values the hazard function receives. The value of the hazard function is not a probability, but it is an indicator of the risk of experiencing the event (George et al. 2014). We will present the shape of survival and hazard functions for the chosen survival model in “Descriptive analysis” section.

This study modelled time to the next engagement in online betting. We employed daily panel data which comprised repeated observations of bettors’ participation in betting and the outcome of each day’s betting. Thus, the data contained multiple events (betting on a particular day) for a large majority of bettors (around 80%) and the outcome of each event varied for each bettor. Since our focus was to explore how betting outcome variables, such as being a *loser on last betting day* or *losses on last betting day,* predict the time to the next event (betting day), we chose to employ the accelerated failure time model (AFT). The main benefit of the AFT model is where events (here betting outcomes) are of variable size (Bradburn et al. 2003).

The use of AFT models requires making an assumption of the statistical distribution which survival times follow. The usual distributional assumptions include exponential, Weibull, generalized gamma, log-normal, and log-logistic. These are used in place of the normal distribution because event times are positively valued and generally have a skewed distribution, making the symmetric normal distribution a poor choice for fitting the data closely (George et al. 2014). We compared the performance of models with diverse distributional assumptions using the Akaike information criterion (AIC). We decided to work with the log-logistic distribution because it had the best fit to the data.

Following the notation expressed in Lee and Wang (2013) who represnted the log-logistic AFT model for logarithm of survival time $T_{i}$ for individual i as

1 $$Log_{{}} T_{i} = a_{0} + \sum\limits_{k = 1}^{p} {a_{k} } x_{ki} + \sigma \varepsilon_{i} ,$$

where $$x_{k}$$, k = 1,…,p, are the covariates, $$a_{k}$$, k = 0,1,…,p the coefficients, *σ*(*σ* > 0) is an unknown scale parameter and $$\varepsilon_{i}$$, the error term. In principle, the parameter estimates in model (1) have a similar interpretation as in the multiple covariate log-linear (OLS) regression model. Holding other covariates constant, an increase in the covariate *j* predicts a percentage change in the survival time, calculated as (exp($$\overset{\lower0.5em\hbox{$\smash{\scriptscriptstyle\frown}$}}{a}_{j}$$) − 1) * 100. When the exponent of the covariate *j*’s regression estimate (i.e. exp($$\overset{\lower0.5em\hbox{$\smash{\scriptscriptstyle\frown}$}}{a}_{j}$$)) is more than 1, an increase in the value of the covariate predicts a longer survival time. In contrast, if the exponent of the covariate *j*’s regression estimate is less than 1, an increase in the value of the covariate predicts that the event occurs faster than expected.

## Results

### Descriptive Statistics

Table 1 reports descriptive statistics of the data. Since the dependent and focus variables may vary over time, we represented the bettor’s average betting day’s figures for these attributes. We found that the average bettor was a 51 year-old male who resides in an urban area, who participated in betting about once in every four days and ended up losing on 70% of his betting days. The last rows show statistics for the latter 15-day period. The average betting volume per day was 43€, the average net loss per day was 12€, and bettors participated in betting six times on average during the observation period.

**Table 1:** Descriptive statistics

	Mean	Std.	Median	p10	p90
*Dependent variables*					
Time to next betting day (in days)	3.60	2.52	2.80	1.17	7.00
*Focus variables*					
Loser on last betting day	0.71	0.15	0.74	0.50	0.88
Losses on last betting day	0.32	12.71	0.46	− 0.47	1.63
Winnings on last betting day	0.08	15.57	0.00	− 0.73	0.24
Age × loser on last betting day	41.98	22.52	48.00	0	65.00
Experience × loser on last betting day	0.41	0.49	0.00	0.00	1.00
*Control variables*					
Age (years)	50.76	12.87	52.00	32.00	66.00
Experience (1 = for high experience)	0.50	0.50	1.00	0.00	1.00
Female (1 = for female)	0.15	0.36	0.00	0.00	1.00
Urban residence (1 = for urban area)	0.76	0.43	1.00	0.00	1.00
Past frequency	0.39	0.27	0.33	0.07	0.80
*Other variables*					
Betting volume in a day (€)	43.43	101.44	19.00	3.75	90.50
Net return of betting day (€)	− 12.01	46.50	− 6.74	− 36.89	4.40
Played days	6.04	3.79	5.00	2.00	12.00

The number of bettors is 9151

### Descriptive Analysis

**Fig. 1:**
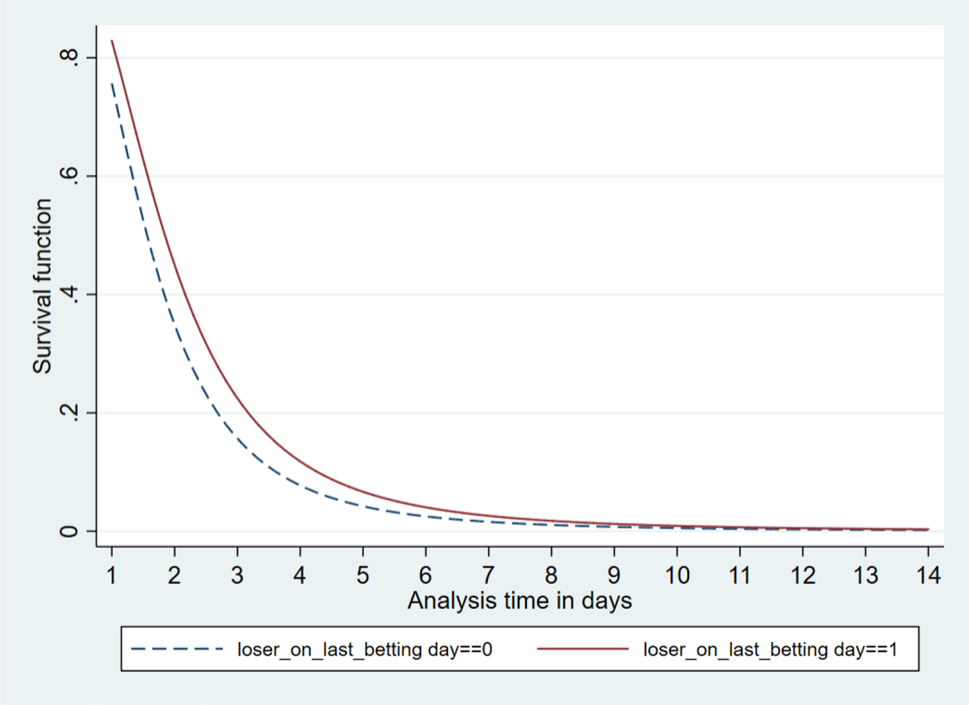
Survival function

**Fig. 2:**
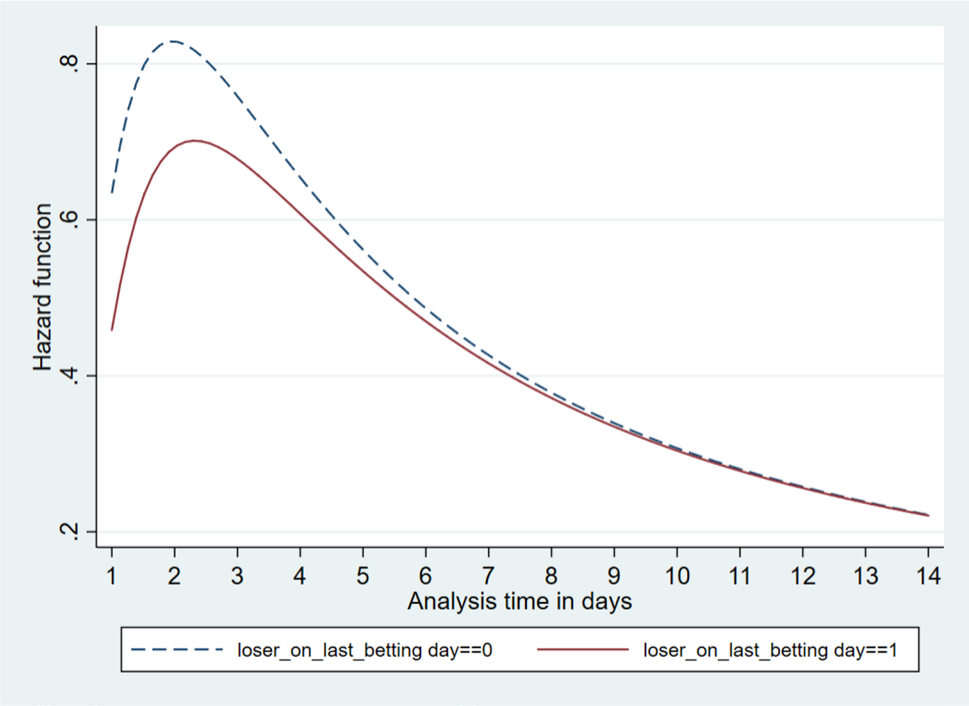
Hazard function

Since the aim was to provide information on how being a loser on the last betting day predicts the duration to the next betting day, we present these functions by assigning the values 0 and 1 to the covariate *loser on last betting day*. Thus, to illustrate possible behavioural differences between bettors who were losers on the last betting day and bettors who ended up even or winning on the last betting day, we had separate functions for these bettor groups. We produced Figs. 1 and 2 using Model 1 in Table 2.

**Table 2:** Survival regression results

	Model 1		Model 2		Model 3	
	Coef.	Std.	Coef.	Std.	Coef.	Std.
*Focus variables*						
Loser on last betting day	0.167***	0.006	0.191***	0.009	0.295***	0.027
Losses on last betting day	− 0.34 × 10^−4^	0.77 × 10^−4^	− 0.31 × 10^−4^	0.78 × 10^−4^	− 0.37 × 10^−4^	0.77 × 10^−4^
Winnings on last betting day	0.30 × 10^−4^	0.29 × 10^−4^	0.30 × 10^−4^	0.29 × 10^−4^	0.28 × 10^−4^	0.29 × 10^−4^
*Control variables*						
Age (years)	− 0.001**	0.37 × 10^−3^	− 0.001**	0.37 × 10^−3^	0.001	0.001
Age × loser on last betting day					− 0.002***	0.001
Female (1 = for female)	0.041***	0.013	0.041***	0.013	0.041***	0.013
Experience (1 = for exp.)	− 0.003	0.010	0.033***	0.014	0.033**	0.014
Experience × loser on last betting day			− 0.045***	0.012	− 0.045***	0.012
Urban residence (1 = for urban area)	− 0.022**	0.011	− 0.022**	0.011	− 0.023**	0.011
Past frequency	− 1.324***	0.017	− 1.324***	0.017	− 1.322***	0.017
Constant	1.261***	0.024	1.241***	0.024	1.160***	0.031
Day of week dummies	Yes		Yes		Yes	
Log likelihood	82 948.011		82 939.543		82 932.668	
Number of obs.	55 313		55 313		55 313	
Number of bettors	9151		9151		9151	

Statistical significance: ***significant at 1%, **significant at 5%

The y-axis in Fig. 1 represents the probability for the average bettor to abstain from betting beyond a specific time given on the x-axis. For instance, we found an 80% chance that a bettor has not returned to betting after 1 day and a 10% chance that a bettor has not bet again after 4 days from his or her previous engagement. Moreover, we found that bettors who had ended up losing on their last active betting day (i.e. *loser on last betting day* = 1) are more likely to stay away from betting in the following few days.

The hazard function is an indicator of the risk of having had another betting experience by each time-since-previous-betting-day, as indicated on the x-axis. We found that the shape of the hazard function in Fig. 2 increased at first, peaked at around 2 days after the last activity, and then started to decrease. We found that the probability that there has been another engagement in betting was more than 70% for losers on the last betting day and more than 80% for bettors who had ended up breaking even or winning on their preceding active betting day. In general, this shape of hazard function supported using a log-logistic, log-normal or generalised gamma distribution assumption (e.g. Bradburn et al. 2003).

### The Day of the Week Effect on Betting Participation

The main betting days in the Finnish horse race betting are Wednesdays and Saturdays. During the data period, the main track in the country (Vermo, Helsinki) organised a popular pool game on Wednesday and another popular pool was offered at changing venues on Saturday.

**Fig. 3:**
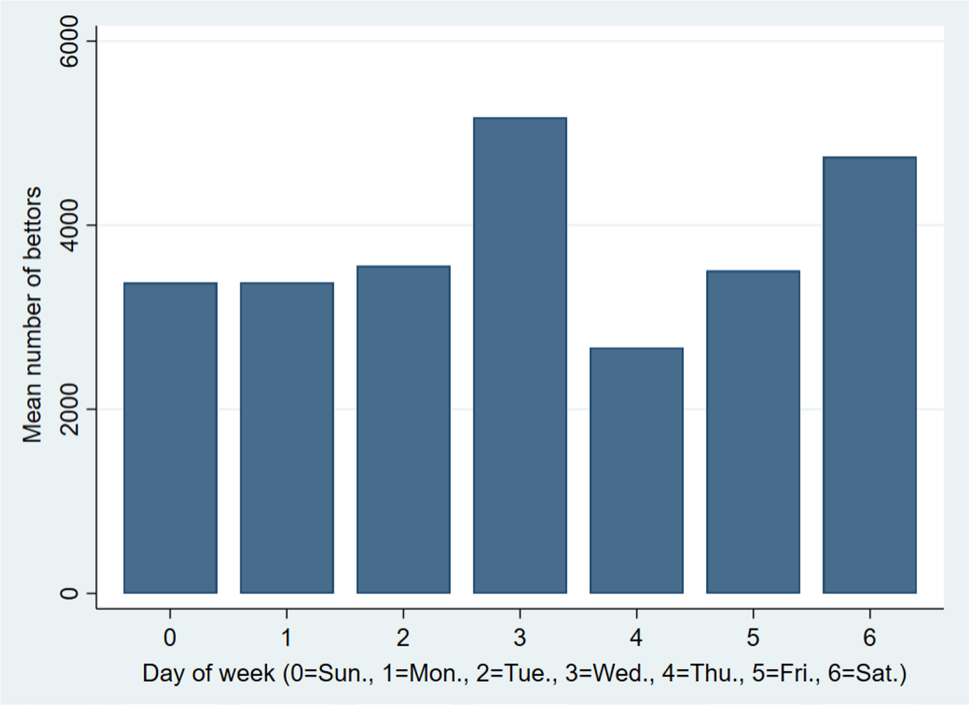
The average betting activity on each day of the week during the second period

Figure 3 provides information on the average number of bettors on each day of the week. During the data period Wednesdays (with the mean 5170) were the most popular betting days, whereas on the least popular betting day, Thursday (with the mean of 2670), the number of bettors nearly halved from the Wednesday’s numbers. Given the systematic differences in the number of bettors between the days of the week, we included the day of the week dummy variables in our regression model.

### Regression Results

This section examined the association between the time to the next betting activity and independent variables using survival regression. In particular, we were interested in how the previous betting day’s outcomes (i.e. *loser on last betting day, losses* or *winnings on last betting day*) predicted the timing of the next betting activity. In addition, we employed an interaction term between *age* and *loser on last betting day* to capture whether an individual’s reaction to losing varied with age. An interaction term with experience allowed a similar test based on how experienced bettor an individual was. Thus, Table 2 provides results of three survival regression models, Model 1 was the baseline model without interaction terms, Model 2 included only interaction terms between *experience* and l*oser on last betting day* and Model 3 included both interaction terms (i.e. *experience* × *loser on last betting day* and *age* × *loser on last betting day*. The focus variables were described in more detail in “Sample” section.

The control variables in Table 1 provided the following information: (1) on average, females kept away from betting after the last betting activity around 4% longer than males, (2) urban bettors participated in betting around 2% sooner after the last betting activity than bettors from rural areas.^2^ On the other hand, untypically high losses or wins in the last betting session were not significant predictors of the amount of time to the next betting event. We also found that the *past frequency* of betting was a strong predictor of current betting frequency. Thus, bettors who had a high frequency in the first period were also likely to have a high number of betting days in the second period and thus return to wagering faster than bettors with a low frequency in the past.

Perhaps, the most interesting findings in Table 1 related to the focus variable *loser on last betting day* in the baseline model and when it was moderated by the interaction terms.^3^ First, we found that *loser on last betting day* is a significant and positive predictor of the amount og time to the next betting day in all models. Thus, we found that bettors who broke even or won returned to betting faster. Second, the coefficient on *experience* was negative and non-significant in Model 1. However, when we included the interaction term *experience* × *loser on last betting day* in Model 2 we found: (1) *experience* was now significant and turned positive, (2) the interaction term was negative and significant. Thus, we found that bettors with high experience were less affected by having a losing day, since they re-entered betting faster after a losing day than bettors with low experience. Thirdly, the coefficient on *age* was negative and significant in Model 1. However, when we included also the interaction term between *age* and *loser on last betting day* in Model 3, we found the following: (1) *age* was no longer significant, (2) *age* × *loser on last betting day* was negative and significant. This implies that there was a difference in how younger and older bettors reacted to a losing day. We found that older bettors tended to return to betting sooner after losses than younger bettors did.

Next we provide some numerical examples on how *experience*, *age* and *loser on last betting day* are linked to the time to the next entry to gambling in Model 3 while keeping other covariates constant. For instance, consider a 50-years-old bettor who was a loser on the last betting day. For a low experience bettor, our model predicted a 27.8% and for a high experience bettor a 26.2% longer time to the next betting day after a losing betting day than otherwise.^4^ In practice, if a bettor’s *past frequency* was once in every 4 days, after a losing day he or she re-entered to betting on the fifth day. Next, we illustrate how *age* predicts time to next event. Consider an experienced 30 year old bettor, whose previous betting day was unprofitable. The model predicted that this bettor would return betting 28.8% later than otherwise. Suppose we had another experienced bettor, who was 70 year old and also ended up losing during the last betting event. The model predicts that this older bettor would stay away from betting only 23.7% longer than otherwise. Overall, we perceived that bettors with different *experience* or *age* reacted differently to being a *loser on last betting day*, but the magnitude of these differences were minor. In conclusion, *loser on last betting day* in our models predicted that the average bettor stayed away from betting longer after a losing day than after a winning day, regardless of his or her age and the level of experience.

## Discussion

This paper added to the small but growing research area which uses player account data to study how past gambling outcomes affect current gambling consumption. We focused on how the outcome of a betting day influenced the amount of time to the next betting event among online horse bettors. We employed survival regression, which has not been commonly used in the field of gambling studies.

We found that the average bettor stayed away from betting around 27% longer after a losing day than otherwise. Our result also expanded the study of Suhonen and Saastamoinen (2018), who studied how a prior outcome of betting affects current consumption during a session, also using Finnish online horse race betting data. They found that during a session a bettor tends to make less risky betting choices in the next race if the bettor is losing. Thus, we found a reduction in gambling activity for the average bettor in between gambling sessions. This finding is consistent with the majority of the previous literature (i.e. Ma et al. 2014; Forrest and McHale 2016). We also found that this pattern was stronger for younger bettors and for relative newcomers to horse betting.

We found that previous betting frequency is a strong predictor of current betting frequency for the average bettor. This is consistent with the previous literature (i.e. Ma et al. 2014; Forrest and McHale 2016) and indicates that online horse race bettors exhibit a repeated behavioural pattern or habit. Additionally, we found that untypically high losses or wins for a person on the last betting day did not predict the amount of time to the next gambling visit for the average player. Thus, we found that whether the average bettor wins or loses in the previous betting session untypically large amount, he or she did not change his or her frequency of participation in gambling. Alternatively, our result suggest that finishing ahead (or breaking even) in gambling is a decisive factor for a bettor, whereas the amount won is not. This is a surprising result and requires, perhaps, further research, since Forrest and McHale (2016) reported the opposite with longer data period from casino gambling.^5^

To overcome the limitations of the study, future studies could employ data sets that include all betting types and cover longer periods of time than the one month used in this study. The longer data period would provide more comprehensive analysis for at least two reasons. First, with the 30-day data used we were unable to reliably measure typical losses. Typical losses were measured during the first 15-day period, which may be subject to noise at least for the less frequent bettors. Second, instead of providing only the results for the average bettor, we could be able to provide estimation results at an individual-level as Narayanan and Manchanda (2012) and Forrest and McHale (2016) have done. This would provide more detailed information on diverse bettor profiles, particularly of bettors who increase their risk in gambling after losses. Moreover, using longer data periods in the future studies could enable shedding light on the prevalence of loss-chasing behaviour in different gambling forms.

In conclusion, we found that the average bettor stayed away longer from betting after a losing day than otherwise. This was an important finding, since previous studies have found that bettors tended to reduce their stakes after a losing session. We expanded this literature by finding a reduction pattern in betting activity for the average bettor after a losing session with a time- related metric in online gambling. Thus, we provided information on the representative player on a question that all problem gambling screens include: whether a respondent returns for another day to try to win back his or her losses. In addition, our findings in a sense validated that loss-chasing behaviour is clearly unusual in a population of gamblers.

## Footnotes

1 Since the aim was to model the average player’s reaction to recent losses, we did not exclude bettors whose typical outcome of betting was now generated by a single observation (i.e., those who had only one observation during the first period). Excluding these bettors, would have lead us to follow only more ‘regular’ bettors. As a robustness check, we also estimated models employing only regular bettor data and the main results were qualitatively similar to those reported here.

2 These are calculated as [exp(0.041)) − 1] × 100 = 4.19% and [exp(−0.022)) − 1] × 100 = − 2.18%.

3 We also experimented with including an interaction term between *female* and *loser on last betting day*, but since it was non-significant we did not include it in our model. This implies that there is no gender difference in how individuals modify behaviour in response to having lost money on the most recent betting day.

4 This is calculated as [exp(0.295 + (0.033–0.045) + 50(0.001–0.002)) − 1] × 100%.

5 As a robustness check, we also used cumulative losses and wins, but the result remains the same. Given that, this study does not use the weighting procedure of attributes presented in Forrest and McHale (2016).

## References

[CR1] Bradburn MJ, Clark TG, Love SB, Altman DG (2003). Survival analysis part III: Multivariate data analysis—Choosing a model and assessing its adequacy and fit. British Journal of Cancer.

[CR3] Cleves M, Could W, Gutierrez RG, Marchenko YV (2010). An introduction to survival analysis using Stata.

[CR4] Forrest, D., & McHale, I. (2016). Tracked play on B1 gaming machines in British casinos. The responsible Gambling Trust-report. Retrieved from https://about.gambleaware.org/media/1300/tracked-play-revision-26-6-16.pdf. Accessed 16 Sept 2020.

[CR5] George B, Seals S, Aban I (2014). Survival analysis and regression models. Journal of Nuclear Cardiology.

[CR6] Ishak JK, Kreif N, Muszbek ABN (2013). Overview of parametric survival analysis for health-economic applications. Pharmaco Economics.

[CR7] Jolley B, Mizerski R, Olaru D (2006). How habit and satisfaction affects player retention for online gambling. Journal of Business Research.

[CR8] Kainulainen T (2019). A new measure of risk-taking in gambling. International Gambling Studies.

[CR9] Kiefer NM (1988). Economic duration data and hazard functions. Journal of Economic Literature.

[CR11] LaBrie RA, Kaplan SA, LaPlante DA, Nelson SE, Shaffer HJ (2008). Inside the virtual casino: A prospective longitudinal study of actual Internet casino gambling. European Journal of Public Health.

[CR12] LaBrie RA, LaPlante DA, Nelson SE, Schumann A, Shaffer HJ (2007). Assessing the playing field: A prospective longitudinal study of Internet spots gambling behavior. Journal of Gambling Studies.

[CR13] LaPlante DA, Kleschinky JH, LaBrie RA, Nelson SE, Shaffer HJ (2009). Sitting at the virtual poker table: A prospective epidemiological study of actual Internet poker gambling behavior. Computers in Human Behavior.

[CR14] Lee ET, Go OT (1997). Survival analysis in public health research. Annual Review of Public Health.

[CR15] Lee ET, Wang JW (2013). Statistical methods for survival analysis.

[CR16] Ma X, Kim SH, Kim SS (2014). Online gambling behavior: The impacts of cumulative outcomes, recent outcomes and prior use. Information System Research.

[CR17] Mas-Verdu F, Ribeiro-Soriano D, Roig-Tierno N (2015). Firm-survival: The role of incubators and business characteristics. Journal of Business Research.

[CR18] Mills M (2011). Introducing survival and event history analysis.

[CR19] Narayanan S, Manchanda P (2012). An empirical analysis of individual level casino gambling behavior. Quantitative Marketing and Economics.

[CR20] Plank SB, DeLuca S, Estacion A (2008). High school dropout and the role of career and technical education: A survival analysis of surviving high school. Sociology of Education.

[CR21] Saastamoinen J, Suhonen N (2018). Does betting experience matter in sequential risk taking in horse race wagering?. Economics and Business Letters.

[CR22] Suhonen N, Kainulainen T (2016). Ravivedonlyöjien verkkopelaaminen Suomessa. Erot kulutus- ja pelikäyttäytymisessä sukupuolen ja iän mukaan (Online horse race betting in Finland. An empirical study on age and gender differences in consumer and betting behaviour). Yhteiskuntapolitiikka.

[CR23] Suhonen N, Saastamoinen J (2018). How do Prior gains and losses affect subsequent risk taking? New evidence from Individual-level horse race bets. Management Science.

[CR24] Suhonen N, Saastamoinen J, Kainulainen T, Forrest D (2018). Is timing everything in horse betting? Bet amount, timing and bettors’ returns in pari-mutuel wagering markets. Economics Letters.

